# Unbiased estimation of an optical loss at the ultimate quantum limit with twin-beams

**DOI:** 10.1038/s41598-018-25501-w

**Published:** 2018-05-09

**Authors:** Elena Losero, Ivano Ruo-Berchera, Alice Meda, Alessio Avella, Marco Genovese

**Affiliations:** 1 INRIM, Strada delle Cacce 91, 10135 Torino, Italy; 2 DISAT, Politecnico di Torino, Corso Duca degli Abruzzi 24, 10129 Torino, Italy; 3 INFN Sezione di Torino, via P. Giuria 1, 10125 Torino, Italy

## Abstract

Loss measurements are at the base of spectroscopy and imaging, thus permeating all the branches of science, from chemistry and biology to physics and material science. However, quantum mechanics laws set the ultimate limit to the sensitivity, constrained by the probe mean energy. This can be the main source of uncertainty, for example when dealing with delicate systems such as biological samples or photosensitive chemicals. It turns out that ordinary (classical) probe beams, namely with Poissonian photon number distribution, are fundamentally inadequate to measure small losses with the highest sensitivity. It is known that quantum-correlated pair of beams, named “twin-beam state”, allows surpassing this classical limit. Here we demonstrate they can reach the ultimate sensitivity for all energy regimes (even less than one photon per mode) with the simplest measurement strategy. One beam of the pair addresses the sample, while the second one is used as a reference to compensate both for classical drifts and for fluctuation at the most fundamental quantum level. This capability of selfcompensating for unavoidable instability of the sources and detectors allows also to strongly reduce the bias in practical measurement. Moreover, we report the best sensitivity per photon ever achieved in loss estimation experiments.

## Introduction

The measurement of changes in intensity or in phase of an electromagnetic field, after interacting with matter, is the most simple and effective way to extract relevant information on the properties of a system under investigation, whether a biological sample^1,2^ or a digital memory disc^3^. Intensity measurements enable absorption/transmission estimation, the base of imaging and spectroscopy, pervasive and fundamental techniques in all science fields, from chemistry^4^ to material science^5^ and physics^6^. They are routinely employed in biomedical analysis^7–9^, as well as in atmospheric^10–12^ and food sciences^13,14^.

However, the optical transmission losses experienced by a probe beam while interacting with a system cannot be determined with arbitrary precision, even in principle. Quantum mechanics establishes fundamental bounds to the sensitivity^15–18^, which is limited, in general, by the mean energy of the probe, or, equivalently, by its mean number of photons. This is in accordance to the intuitive idea that gaining the perfect knowledge on a system would require an infinite amount of physical resources.

The lower bound to the uncertainty, when restricted to the use of classical probe states, coincides with the one achieved by a coherent state, ${{U}}_{{coh}}\simeq {\mathrm{[(1}-{\alpha })/\langle {n}_{P}\rangle ]}^{\mathrm{1/2}}$^17^, where 〈*n*_*P*_〉 is the mean number of photons of the probe and 0 ≤ *α* ≤ 1 is the loss of the sample. Indeed, this limit can be obtained in practice by any probe beam exibiting Poissonian photon statistics, as a laser beam (described theoretically by a coherent state) or even a thermal source like LEDs or incandescent light bulbs in the limit of extremely low photon number per mode. Note that the uncertainty depends on the loss parameter, and can be arbitrary small only in the asymptotic limit of high losses. For a faint loss, $\alpha  \sim 0$, one retrieves the expression *U*_*snl*_ = 〈*n*_*P*_〉^−1/2^, usually referred as to “shot-noise-limit” (SNL).

Without restriction on the probe state, it has been shown^18,19^ that the ultimate quantum limit (UQL) in the sensitivity for a single mode interrogation of the sample is ${{U}}_{{uql}}\simeq \sqrt{\alpha }\,{{U}}_{{coh}}$, which scales much more favourably than the classical bound for small losses, a region which is particularly significant in many real applications. It is worth noting that the use of quantum states does not improve the uncertainty scaling with the number of particles. This is different from what happens in phase shift estimation, in which a sensitivity scaling proportional to 〈*n*_*P*_〉^−1^ is reachable in ideal situations^15,16^, the so called “Heisenberg limit”. The fundamental difference is that phase shift is a unitary operation, preserving the purity of the state, while a loss is intrinsically non unitary. A loss can be represented as the action of a beam splitter that mixes up the probe state in one port with the vacuum state in the other port, basically spoiling quantum features such as entanglement, which is necessary to approach the Heisenberg limit^16^.

It is known that single mode squeezed vacuum reaches *U*_*uql*_ for small losses, $\alpha  \sim 0$, and small number of photons $\langle {n}_{P}\rangle  \sim 0$^18^. Fock states |*n*〉, having by definition a fixed number of photons, approach *U*_*uql*_ unconditionally, i.e. for all value of *α*, but they cannot explore the regime of 〈*n*_*P*_〉 < 1^19^. The optimal performance of Fock states can be understood by considering that a loss can be easily estimated by comparing the number of photons of the probe before and after the interaction with the sample. The perfect knowledge of the photon number of the unperturbed Fock state allows one to detect better small deviations caused by the sample, which would remain hidden in the intrinsic photon number fluctuation of Poissonian distributed sources.

However it is challenging to produce experimentally true Fock states. A reasonable approximation of a Fock state with *n* = 1 are the heralded single photons produced by spontaneous parametric down conversion (SPDC)^20,21^. In this process photons are always emitted in pairs with low probability, but one can get rid of the vacuum component since the detection of one photon of the pair heralds the presence of the other one. This scheme has been demonstrated recently for quantum enhanced absorption measurement both with post-selection of the heralded single photons^22^ and, more remarkably, with selection performed by active feed-forward enabled by an optical shutter^23^.

Also quantum correlations of twin-beam (TWB) state have shown the possibility of sub-SNL sensitivity in absorption/transmission measurement^24–31^, quantum enhanced sensing^32–35^, ghost imaging^36^, quantum reading of digital memories^37^ and plasmonic sensors^38,39^. TWB states can be generated by SPDC^40^ as well as by four wave mixing in atomic vapours^41–44^, and expose a high level of quantum correlation in the photon number fluctuations between two corresponding modes, for example two propagation directions or two wavelengths. Even if super-Poissonian noise characterizes the photon distribution in one mode, the fluctuations are perfectly reproduced in time and space in the correlated mode. Sub-shot noise correlation of this state has been experimentally demonstrated both in the two-mode case^45–49^ and in the case of many spatial modes detected in parallel by the pixels of a CCD camera^50–52^. The exploitation of spatially multimode non-classical correlation has been proposed for high sensitivity imaging of distributed absorbing object^53^ and a proof of principle of the technique has been reported by Brida *et al*. in^28^. Recently our group has realized the first wide-field sub-SNL microscope^30^, providing 10^4^ pixels images with a true (without post-selection) significant quantum enhancement, and a spatial resolution of few micrometers. This represents a considerable advancement towards a real application of quantum imaging and sensing.

The common idea behind these works is that the random intensity noise in the probe beam addressed to the sample can be known by measuring the correlated (reference) beam and subtracted. Note that the two-beams approach is extensively used in standard devices like spectrophotometers, where a classical beam is split in two by a beam splitter and one beam is used to monitor the instability of the source and detectors and to compensate for them. This is particularly effective in practical applications, since unavoidable drifts in the source emission or detector response would lead to strong bias, especially in the estimation of small absorptions. However, in classical correlated beams (CCB) generated in this way, only the super-Poissonian component of the fluctuations is correlated (sometimes called classical “excess noise”), whereas the shot noise remains uncorrelated and cannot be compensated. Therefore TWB represent the natural extension to the two-beam approach to the quantum domain, promising to be especially effective for small absorption measurement and when low photon flux is required.

It has been theoretically demonstrated^54^ that using TWB for loss estimation the UQL is in principle attainable; nevertheless the existence of an experimental estimator fit for this purpose is still an open question, as it is its explicit expression.

Here, we show that the answer to this question is unconditionally positive considering TWB generated by SPDC process, for all the energy regime and all values of the loss parameter *α*. Therefore, TWB overcome the limitations of both single mode squeezed vacuum and Fock states, representing the practical best choice for pure loss estimation. We prove this result by an operative approach: we consider a specific and simple measurement strategy, proposed for the first time by Jakeman and Rarity^24^, that is to evaluate the ratio between the photon number measured in the probe and in the reference beam. In the ideal lossless detection case this is sufficient to reach the ultimate quantum limit. Taking into account for experimental imperfections, we derive the uncertainty advantage of the twin-beam with respect to the single classical beam (SCB) and to the CCB case in terms of experimental parameters related to the “local” photon statistics of the two beams separately, and the amount of non-classical correlation of the joint photon number statistics.

In a recent work^27^, a different optimized estimator which allows improving the sensitivity in case of strong non-ideal detection efficiencies has been proposed. The drawback is that this method requires the accurate and absolute characterization of the measurement apparatus, in particular the absolute values of the quantum efficiencies of the detectors and of the excess noise of the source. This aspect places a strong practical limitation, because the determination of quantum efficiency, especially at the few photon level, with uncertainty less than 10^−3^ is extremely challenging, limiting the overall accuracy of the method; then, instabilities could also affect the measurement. We show that the simplest estimator in ref.^24^ behaves almost as good as the optimized one for relatively high values of the efficiencies (the condition of our experiment), but it requires the weakest assumptions on the stationarity of the system and does not require absolute value of any parameter.

Finally we perform the experiment, measuring intensity correlations in the far field of multi-mode parametric down conversion by a standard low noise and high efficiency CCD camera. For a sample loss of $\sim \mathrm{2 \% }$, we report an experimental quantum enhancement in the estimation uncertainty of 1.51 ± 0.13 with respect to the single beam classical probe and of 2.00 ± 0.16 compared to the classical two-beam approach, when the same mean energy of the probe and the same detection efficiency are considered.

## Theory

**Figure 1:**
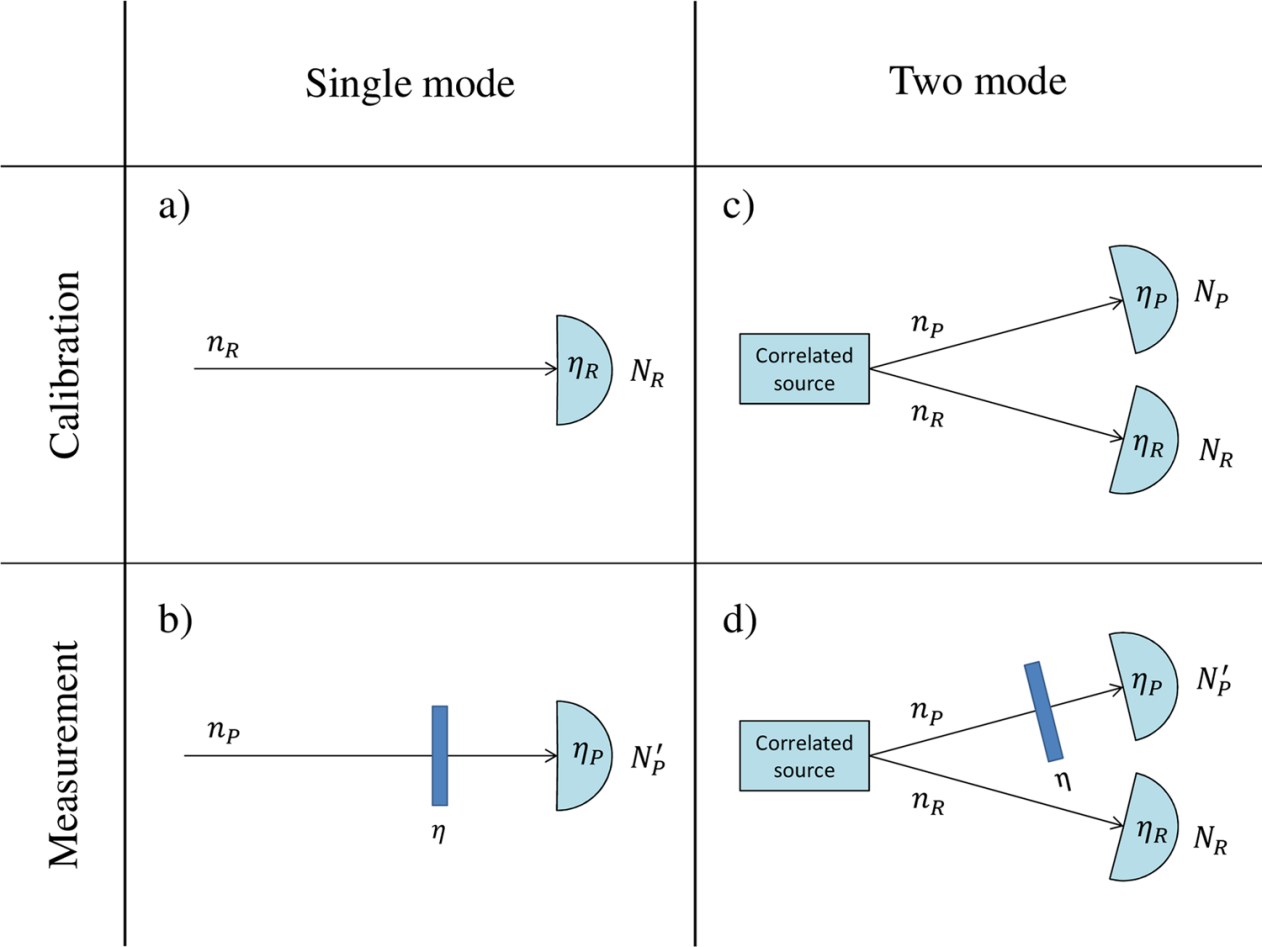
Two possible schemes to estimate the absorption coefficient *α*. In the single-mode case (**a**) and (**b**) there is no correlation between probe and reference beam, i.e. 〈Δ*N*_*p*_Δ*N*_*R*_〉 = 0 while in the two-mode case (**c**) and (**d**) 〈Δ*N*_*p*_Δ*N*_*R*_〉 ≠ 0. Different possibilities of input states and absorption estimators for both the schemes are discussed in the text.

In practice, an optical loss *α* can be easily measured by comparing the number of photons of the probe ${N}_{P}^{^{\prime} }$ after a lossy interaction, with a reference value *N*_*R*_, which can be evaluated in a previous moment in absence of the sample (Fig. 1a) or by the help of a second beam (Fig. 1d). In particular, one can consider the estimator^24^:

1 $${S}_{\alpha }=1-\gamma \frac{{N}_{P}^{^{\prime} }}{{N}_{R}}.$$

The factor *γ* = 〈*N*_*R*_〉/〈*N*_*P*_〉 should be introduced in case of unbalancing between the mean energy of probe and reference beams and evaluated in a pre-calibration phase of the apparatus (Fig. 1c). A loss is a random process modelled by the action of a beam splitter of transmission 1 − *α*, so that the statistics of the photon counting of the probe beam is modified in this way^40^:

2 $$\langle {N}_{P}^{^{\prime} }\rangle =(1-\alpha )\langle {N}_{P}\rangle ,$$

3 $$\langle {{\rm{\Delta }}}^{2}{N}_{P}^{^{\prime} }\rangle =[{\mathrm{(1}-\alpha )}^{2}({F}_{P}-\mathrm{1)}+1-\alpha ]\,\langle {N}_{P}\rangle .$$

Here *N*_*P*_ is the measured photon number without the sample. Its fluctuation is represented by the Fano factor *F*_*P*_ = 〈Δ^2^*N*_*P*_〉/〈*N*_*P*_〉 ≥ 0 which quantifies the non-classicality of the photon statistics. In particular *F*_P_ < 1 indicates sub-Poissonian noise^55^ and in general the possibility to surpass the SNL.

By expanding the photon number operators in Eq. (1) at the first order around their mean value, the expected value of the estimator becomes^24^:

4 $$\langle {S}_{\alpha }\rangle =\alpha +\mathrm{(1}-\alpha )\frac{\langle {\rm{\Delta }}{N}_{P}{\rm{\Delta }}{N}_{R}\rangle }{\langle {N}_{P}\rangle \,\langle {N}_{R}\rangle }.$$

An unbiased estimation of the loss can be obtained solving the Eq. (4) with respect to *α*. By propagating the uncertainty of the quantities ${N}_{P}^{^{\prime} }$ and *N*_*R*_ on *S*_*α*_, and rewriting the terms using the unperturbed variance 〈Δ^2^*N*_*P*_〉, the quantum expectation value of fluctuation is:

5 $${{\rm{\Delta }}}^{2}{S}_{\alpha }\simeq {U}_{uql,\langle {N}_{P}\rangle }^{2}+\frac{{\mathrm{(1}-\alpha )}^{2}}{\langle {N}_{P}\rangle }\frac{2{\sigma }_{\gamma }}{\gamma }.$$

Note that ${U}_{uql,\langle {N}_{P}\rangle }$ has the form of the UQL but refers to the number of detected photons. Considering the probes photons 〈*n*_*P*_〉 incident on the sample, one has ${U}_{uql,\langle {n}_{P}\rangle }={U}_{uql,\langle {N}_{P}\rangle }\sqrt{{\eta }_{d}}$, where *η*_*d*_ represents the detection efficiency, i.e. the losses experienced after the sample. The most relevant quantity appearing in Eq. (5) is the positive factor:

6 $${\sigma }_{\gamma }=\frac{\langle {{\rm{\Delta }}}^{2}({N}_{R}-\gamma {N}_{P})\rangle }{\langle {N}_{R}+\gamma {N}_{P}\rangle }=\frac{\langle {{\rm{\Delta }}}^{2}{N}_{R}\rangle +{\gamma }^{2}\langle {{\rm{\Delta }}}^{2}{N}_{P}\rangle -2\gamma \langle {\rm{\Delta }}{N}_{P}{\rm{\Delta }}{N}_{R}\rangle }{\langle {N}_{R}+\gamma {N}_{P}\rangle }.$$

In the case of *γ* = 1 it represents the quantifier of the non-classical correlation known as noise reduction factor (NRF), *σ* = *σ*_*γ*=1_, where the bound between classical and quantum correlations is set by *σ* = 1. Thus, the uncertainty is expressed in terms of simple measurable quantities related to the photon number statistics, i.e. the intensity fluctuations. Eq. (5) shows that whenever *γ* = 1 and *σ* = 0 the UQL is retrieved, ${{\rm{\Delta }}}^{2}{S}_{\alpha }(\gamma =1,\,\sigma =0)\simeq {U}_{uql,\langle {N}_{P}\rangle }^{2}$.

In the following we consider different states for the probe and the reference beam to establish the limit to the sensitivity in relevant scenarios.

Let us first focus on the states which do not present correlation between probe and reference (e.g. the measurements on the probe and reference beam are performed in two different moments, refer to Fig. 1 a), b), so that 〈Δ*N*_*P*_Δ*N*_*R*_〉 = 0.

- *Fock states*. It is clear that the only chance for uncorrelated states to achieve the condition *σ*_*γ*_ = 0 and hence the UQL according to Eq. (5) is to have null fluctuation in the photon number both for the reference and probe beam, 〈Δ^2^*N*_*R*_〉 ≡ 〈Δ^2^*N*_*P*_〉 ≡ 0. This means that the state must be the product of two unperturbed Fock states, $|n{\rangle }_{P}\otimes |n{\rangle }_{R}$ detected with unitary efficiency. Thus, as anticipated, Fock states reaches the UQL unconditionally, i.e. for all the value of the parameter, with the only limitation that the mean photon number cannot be arbitrarily small^19^ (i.e. 〈*n*_*P*_〉 ≥ 1).

7 $${{\rm{\Delta }}}^{2}{S}_{\alpha }^{(Fock)}\simeq {U}_{uql,\langle {n}_{P}\rangle }^{2}$$

- *Coherent states*. Let us now consider the state $|coh{\rangle }_{P}\otimes |coh{\rangle }_{R}$, particularly interesting for its simple experimental implementation. In the photon number basis, coherent states have the form $|coh\rangle ={e}^{-\frac{1}{2}\langle n\rangle }{\sum }_{n\mathrm{=0}}^{\infty }\frac{{\langle n\rangle }^{n\mathrm{/2}}}{\sqrt{n!}}|n\rangle$, following the Poissonian photon number distribution *P*_*coh*_(*n*) = *e*^−〈*n*〉^〈*n*〉^*n*^/*n*!, which has the property 〈Δ^2^*n*〉 = 〈*n*〉. Thus, substituting the variances with the mean values in the right hand side of Eq. (6) one get *σ*_*γ*_ = (1 + *γ*)/2, and accordingly:

8 $${{\rm{\Delta }}}^{2}{S}_{\alpha }^{(coh)}\simeq {U}_{uql,\langle {N}_{P}\rangle }^{2}+\frac{{\mathrm{(1}-\alpha )}^{2}}{\langle {N}_{P}\rangle }\frac{1+\gamma }{\gamma }.$$

The lower limit for a pair of coherent states is reached under the condition of $\gamma \gg 1$, i.e. when the reference beam has much more energy than the transmitted probe, and the relative fluctuation on its photon number becomes negligible. In this case ${{\rm{\Delta }}}^{2}{S}_{\alpha }^{(coh)}$ equals the classical lower bound, detection efficiency apart, ${{\rm{\Delta }}}^{2}{S}_{\alpha }^{(coh)}=$ $(1-\alpha )/\langle {N}_{P}\rangle ={\eta }_{d}^{-1}{U}_{coh,\langle {n}_{P}\rangle }^{2}$. In practice, one can also consider an equivalent situation, in which the reference uncertainty has been statistically reduced to a negligible contribution by a long acquisition time in the calibration phase (Fig. 1a), namely a time much longer than the one used for the measurement of the probe beam in presence of the sample (Fig. 1b). Indeed, replacing the variable *N*_*R*_ with its mean value 〈*N*_*R*_〉 in the definition of *S*_*α*_ and of *σ*_*γ*_ in Eq. (6) leads to the an identical limit of the sensitivity.

More in general, it is convenient to rewrite the noise reduction factor for uncorrelated states in terms of the measurable Fano factor of each beam in absence of the sample, i.e. *σ*_*γ*_ = (*F*_*R*_ + *γF*_*P*_)/2. With this substitution, Eq. (5) becomes:

9 $${{\rm{\Delta }}}^{2}{S}_{\alpha }^{(unc)}\simeq {U}_{uql,\langle {N}_{P}\rangle }^{2}+\frac{{\mathrm{(1}-\alpha )}^{2}}{\langle {N}_{P}\rangle }(\frac{1}{\gamma }{F}_{R}+{F}_{P}).$$

The measured Fano factors account for the statistics of light sources and for transmission inefficiency and detection losses. If 0 ≤ *η*_*j*_ ≤ 1(*j* = *P*, *R*) is the overall channel efficiency, including the detection one *η*_*d*_ and the losses between the source and the sample, the Fano factor can be written as ${F}_{j}={\eta }_{j}{F}_{j}^{\mathrm{(0)}}+1-{\eta }_{j}$, where ${F}_{j}^{\mathrm{(0)}}$ refers to the one of the unperturbed state of the source. As expected, detection losses deteriorate the non classical signature of the probe and reference beams, preventing the real possibility to reach the UQL even with Fock states.

Considering now joint states where a correlation between probe and reference is present, i.e. 〈Δ*N*_*P*_Δ*N*_*R*_〉 ≠ 0 (Fig. 1c,d) we have:

- *TWB state*. Two mode twin beam state, generated by SPDC, is represented by the following entangled state in the photon number basis {|*n*〉}^56^:

10 $$|TWB{\rangle }_{PR}=[\langle n\rangle +{1]}^{-1/2}\sum _{n=0}^{{\rm{\infty }}}{[\frac{\langle n\rangle }{\langle n\rangle +1}]}^{n/2}|n{\rangle }_{P}|n{\rangle }_{R}.$$

The two modes, separately, obey to a thermal statistics each, where 〈Δ^2^*n*〉 = 〈*n*〉(1 + 〈*n*〉). However, they are balanced in the mean energy, 〈*n*_*P*_〉 = 〈*n*_*R*_〉, and their fluctuations are perfectly correlated, 〈Δ*n*_*P*_Δ*n*_*R*_〉 = 〈Δ^2^*n*〉. This leads to *γ* = 1 and *σ* = 0, thus demonstrating that TWB detected with unitary efficiency reaches the *U*_*uql*_, according to Eq. (5). Note that this result is independent on the value of the parameter *α* and on the energy of the probe beam which can contain less than one photon per mode on average. Indeed, this is usually the case in experiments.

- *Classical correlated beams (CCB)*. Let us consider a bipartite correlated state produced by a unitary splitting of a single beam. Given a splitting ratio 0 ≤ *τ* ≤ 1, it turns out that the statistics of the two out-coming beams, the probe and the reference, is characterized by *γ* = *τ*^−1^ − 1 and *σ*_*γ*_ = (2*τ*)^−1^, which are remarkably independent on the photon number distribution of the initial beam. Substituting these values in Eq. (5) leads to the same uncertainty of two uncorrelated coherent beams ${{\rm{\Delta }}}^{2}{S}_{\alpha }^{(CCB)}={{\rm{\Delta }}}^{2}{S}_{\alpha }^{(coh)}$, reported in Eq. (8). It shows that classical correlation can never approach the UQL, and that the lower uncertainty is achieved for a splitting ratio $\tau \simeq 0$ corresponding to a strong unbalancing of beam energies, $\langle {N}_{P}\rangle \ll \langle {N}_{R}\rangle$. Therefore, for the specific measurement strategy considered here and whatever the input state, it is convenient to use a highly populated reference beam and a weak prope beam. This result is in agreement with the behaviour reported by Spedalieri *et al*.^57^ in the complementary situation in which the input state is a thermal one while the measurement strategy is the most general one allowed by quantum mechanics.

Finally, to better understand how losses or excess noise of the source influence the final accuracy in real experiment we note that the parameter *σ*_*γ*_ can be rewritten as ${\sigma }_{\gamma }=\frac{\gamma +1}{2}\sigma +\frac{\gamma -1}{2}({F}_{R}-\gamma {F}_{P})$. In presence of equal losses in both the branches *η*_*R*_ = *η*_*P*_ = *η*, the noise reduction factor, expressed in terms of the ideal unperturbed one *σ*^(0)^, is *σ* = *ησ*^(0)^ + 1−*η*. For the relevant case of a TWB state, it is *F*_*R*_ = *F*_*P*_, *γ* = 1 and *σ*^(0)^ = 0, leading to:

11 $${{\rm{\Delta }}}^{2}{S}_{\alpha ,\eta }^{(TWB)}\simeq {U}_{uql,\langle {N}_{P}\rangle }^{2}+2\frac{{\mathrm{(1}-\alpha )}^{2}}{\langle {N}_{P}\rangle }(1-\eta ).$$

This expression shows how the degradation of the accuracy in presence of losses prevents reaching the UQL in practice.

On the other side, for *γ* = 1, balanced CCB (bCCB) fulfills the lower classical bound *σ*_*γ*_ = *σ* = *σ*^(0)^ = 1, thus using Eq. (5) we obtain:

12 $${{\rm{\Delta }}}^{2}{S}_{\alpha ,\eta }^{(bCCB)}\simeq {U}_{uql,\langle {N}_{P}\rangle }^{2}+2\frac{{\mathrm{(1}-\alpha )}^{2}}{\langle {N}_{P}\rangle }=\frac{\mathrm{(1}-\alpha \mathrm{)(2}-\alpha )}{\langle {N}_{P}\rangle }.$$

Note that in case of bCCB, the accuracy is immune from the detection losses but it is always worse than in the case of TWB reported in Eq. (11).

Up to now we have analyzed the performance of the specific estimator in Eq. (1), showing that it allows reaching the optimal limits both for classical and quantum states, in particular using TWB state the UQL is retrieved. However, other estimators have been considered in literature for absorption measurement with TWB. An interesting alternative is the estimator used in the recent experiment by Moreau *et al*.^27^,

13 $${S}_{\alpha }^{^{\prime} }=1-\frac{{N}_{P}^{^{\prime} }-k{\rm{\Delta }}{N}_{R}+\delta E}{\langle {N}_{P}\rangle },$$

where the weight factor *k* can be determined in order to minimize the uncertainty on ${S}_{\alpha }^{^{\prime} }$, while *δE* is a small correction introduced to render the estimator unbiased. However, *k* and *δE* need to be estimated in a phase of pre-calibration of the apparatus. In particular it turns out that *k*_*opt*_ is a function of the detection efficiencies of the channels and the local excess noise *k*_*opt*_ = *f*(*η*_*P*_, *η*_*R*_, *F*_*P*_, *F*_*R*_) while *δE* depends also from the measured covariance 〈Δ*N*_*P*_Δ*N*_*R*_〉. We have evaluated analytically in the general case, with the only hypothesis of balanced sources, the expected uncertainty of the estimator in Eq. (13) when *k* = *k*_*opt*_. For the sake of simplicity, here we report the expression obtained in case of symmetric statistical properties of the channels, *γ* = 1 and *F*_*P*_ = *F*_*R*_ = *F*:

14 $${{\rm{\Delta }}}^{2}{S}_{\alpha }^{{}^{{\rm{^{\prime} }}}}={U}_{uql,\langle {N}_{P}\rangle }^{2}+\frac{{(1-\alpha )}^{2}}{\langle {N}_{P}\rangle }\sigma (2-\frac{\sigma }{F}).$$

For TWB and lossless detection, the noise reduction factor *σ* is identically null and the UQL is retrieved also with this estimator. Taking into account balanced detection losses, and the common experimental case of a mean photon number per mode much smaller than one, one can substitute in Eq. (14) *σ* = 1 − *η* and $F\simeq 1$. Therefore, the uncertainty becomes:

15 $${{\rm{\Delta }}}^{2}{S}_{\alpha ,\eta }^{^{\prime} (TWB)}={U}_{uql,\langle {N}_{P}\rangle }^{2}+\frac{{\mathrm{(1}-\alpha )}^{2}}{\langle {N}_{P}\rangle }(1-{\eta }^{2}).$$

Comparing the uncertainty in Eq. (15) with the one reported in Eq. (11) makes clear that the estimator ${S}_{\alpha }^{^{\prime} }$ proposed in^27^ performs better than *S*_*α*_, especially when detection losses are considerable.

Finally, in Brambilla *et al*.^53^ it is suggested to measure the absorption by a differential measurement, considering the following estimator:

16 $${S^{\prime\prime} }_{\alpha }=\frac{{N}_{R}-\gamma {N}_{P}^{^{\prime} }}{\langle {N}_{R}\rangle }.$$

For a source producing a pairs of beams with the same local statistical properties, the variance of ${S}_{\alpha }^{^{\prime\prime} }$ can be calculated as:

17 $${{\rm{\Delta }}}^{2}{S}_{\alpha }^{^{\prime\prime} }=\frac{\mathrm{[2(1}-\alpha ){\sigma }_{\gamma }+\alpha +({F}_{R}-\mathrm{1)}{\alpha }^{2}]}{\gamma \langle {N}_{P}\rangle }.$$

However, this choice is not optimal and depends on the value of the measured local statistics: in the best case of unperturbed TWB, in which *σ*_*γ*_ = 0 and *γ* = 1, it approaches *U*_*uql*_ only asymptotically for ${F}_{R}{\alpha }^{2} \sim 0$. In TWB, produced experimentally by SPDC, the statistics of each mode is thermal with a photon number per mode much smaller than one, thus ${F}_{R}\simeq 1$ and the condition reduces to $\alpha  \sim 0$. Conversely, for high value of the estimated losses, $\alpha  \sim 1$, the performance of this estimator is much worse than the one of *S*_*α*_ and ${S}_{\alpha }^{^{\prime} }$.

## Experiment

**Figure 2:**
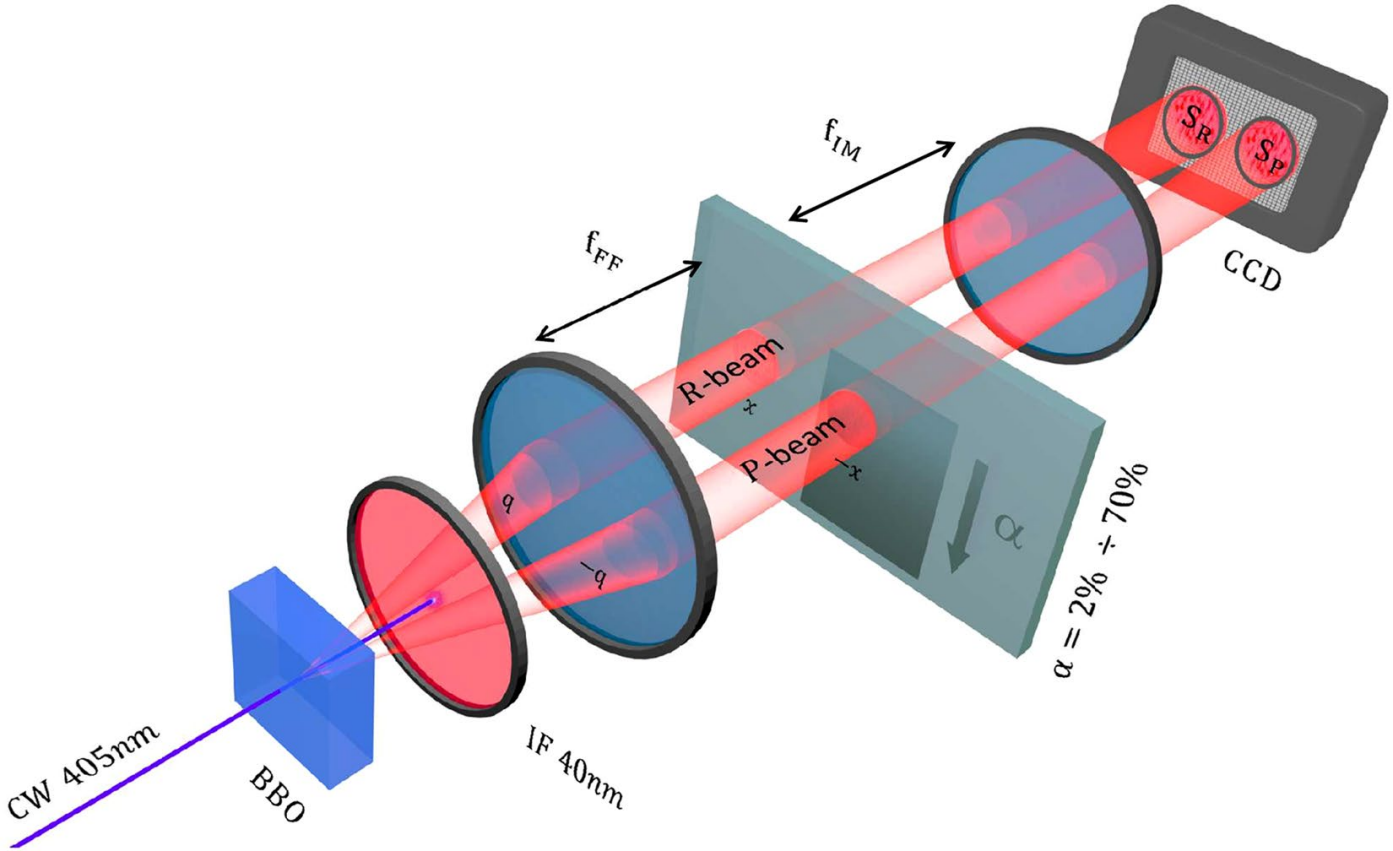
Scheme of our experimental set-up. In the BBO crystal two beams with perfect correlation in the photon number (TWB state) are generated. The probe beam passes trought the sample and is then detected in the *S*_*P*_ region of the CCD, on the contrary the reference beam goes directly to *S*_*R*_, without interacting with the sample. A detailed description of the optical components can be found in the text.

A scheme of the experimental set-up is reported in Fig. 2.

A CW laser-beam (10 mW at *λ*_*pump*_ = 405 *nm*) pumps a 1 cm Type-II-Beta-Barium-Borate (BBO) non linear crystal, where SPDC occurs and two beams with perfect correlation in the photon number are generated. Note that the state |Ψ〉 produced by SPDC process is intrinsically multi-mode and can be expressed, in the plane-wave pump approximation, as a tensor product of two-modes TWB states of the form in Eq. (10) as: |Ψ〉 = ⊗_**q**,*λ*_|*TWB*〉_**q**,*λ*_, where **q** and *λ* are respectively the transverse momentum and the wavelength of one of the two photons produced, while momentum and wavelength of the other photon are fixed by energy and momentum conservation.

The far field of the emission is realized at the focal plane of a lens with *f*_*FF*_ = 1 *cm* focal length. Then a second lens, with *f*_*IM*_ = 1.6 *cm*, images the far field plane to the detection plane. The magnification factor is M = 7.8. The detector is a charge-coupled-device (CCD) camera Princeton Inst. Pixis 400BR Excelon operating in linear mode and cooled down to −70 °C. It presents high quantum efficiency (nominally > 95% at 810 nm), 100% fill factor and low noise (read-noise has been estimated around 5 *e*^−^/(*pixel*⋅*second*)). The physical pixel of the camera measures 13 *μm*, nevertheless, not being interested in resolution, we group them by 24 × 24 hardware binning. This allows us to reduce the acquisition time and the effects of the read-out noise. Just after the crystal an interference filter ((800 ± 20) *nm*, 99% transmittance) is positioned to select only the modes of frequencies around the degeneracy, *λ*_*d*_ = 2*λ*_*pump*_. This choice allows the presence of different spatial modes, in our case we have ${M}_{sp} \sim 2500$ spatial modes impinging on each detection area, *S*_*P*_ and *S*_*R*_, where *P* and *R* subscripts refer to the probe and reference beam, respectively. We integrate the signals in *S*_*R*_ and in *S*_*P*_. The sample consists in a coated glass-slide with a deposition of variable absorption coefficient *α* intercepting the probe beam in the focal plane. We consider values of *α* from 1% to 70%. Finally, in order to check the theoretical model at varying *η*_*R*_ and *η*_*P*_, neutral filters of different absorption can be eventually positioned on the beams path.

The acquisition time of a single frame is set to 100 *ms*, whilst the coherence time of the SPDC process is around 10^−12^ *s*, thus the number of the detected temporal modes is approximatively ${M}_{t} \sim {10}^{11}$. Since in each detection area we register around $\langle {N}_{P}\rangle  \sim 50\cdot {10}^{4}$ photons per frame, it follows that the occupation number of the single spatio-temporal mode is $\mu  \sim 2\cdot {10}^{-9}$ photons/mode. Being $\mu \ll 1$, this implies that the statistic of a single mode is well modeled by a Poissonian statistic: it follows that if only one beam is considered the measurements are shot-noise limited.

However, it is possible to go beyond the shot noise limit exploiting the photon number correlation between pairs of correlated modes. In the plane wave pump approximation with transverse momentum ***q***_*pump*_ = 0, in the far field region any mode with transverse momentum **q** is associated with a single position **x** according to the relation: ${\bf{x}}=\frac{2c{f}_{FF}}{{\omega }_{pump}}{\bf{q}}$, where *c* is the speed of light, *f*_*FF*_ the focal length of the first lens and *ω*_*pump*_ the laser frequency. The exact phase-matching condition for correlated modes ***q***_*P*_ + ***q***_*R*_ = ***q***_*pump*_ = 0 becomes in the far field, for degenerate wavelengths *λ*_*P*_ = *λ*_*R*_ = 2*λ*_*pump*_, a condition on their position: ***x***_*P*_ + ***x***_*R*_ = 0. Under the hypothesis of plane wave pump it is therefore expected that two symmetric pixels of the camera, respect to the pump direction, always detect the same number of photons. For a realistic pump with a certain spread Δ***q*** it follows: ${{\boldsymbol{x}}}_{P}+{{\boldsymbol{x}}}_{R}=0\pm {\rm{\Delta }}{\boldsymbol{x}}=\pm \,\frac{2c{f}_{FF}}{{\omega }_{pump}}{\rm{\Delta }}{\boldsymbol{q}}$. Δ***x*** represents the size in the far field of the so called coherence area, *A*_*coh*_, area in which photons from correlated modes can be collected. Moreover, the non-null frequency bandwidth (about 40 nm in our experiment) determines a further broadening of the spot in which correlated detection events occur. To experimentally measure the size of *A*_*coh*_ the spatial cross-correlation between the two beams can be considered^30^. Its evaluation is important to compare it with the detection area *A*_*det*_ since, to detect a significant level of correlation, it is necessary that *A*_*det*_ ≥ *A*_*coh*_. In our case, integrating on the two regions of interest this condition is fully fulfilled, indeed it holds ${A}_{det}\gg {A}_{coh}$. In general the measured NRF can be modeled as^58^:

18 $${\sigma }_{\gamma }=\frac{1+\gamma }{2}-{\eta }_{R}{\eta }_{coll}\ge \mathrm{0,}$$

where two contributions are present.

- 0 ≤ *η*_*R*_ ≤ 1, the total efficiency of the reference optical path.
- 0 ≤ *η*_*coll*_ ≤ 1, the collection efficiency of correlated photons. This factor represents approximatively the probability that given a detected photon in *S*_*R*_, its “twin” is expected to fall in *S*_*P*_.

**Figure 3:**
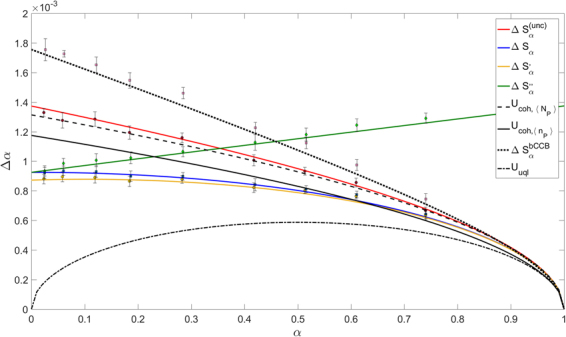
Uncertainty on *α* in function of the mean value of *α*. Four different estimators are considered. Solid lines are the theoretical curves, dashed and dotted lines are the limits corresponding to significant theoretical limits (see text for details), the markers are the experimental data. In this configuration measured efficiencies are *η*_*P*_ = *η*_*R*_ = 0.76 and $\langle {N}_{P}\rangle  \sim 50\cdot {10}^{4}$.

**Figure 4:**
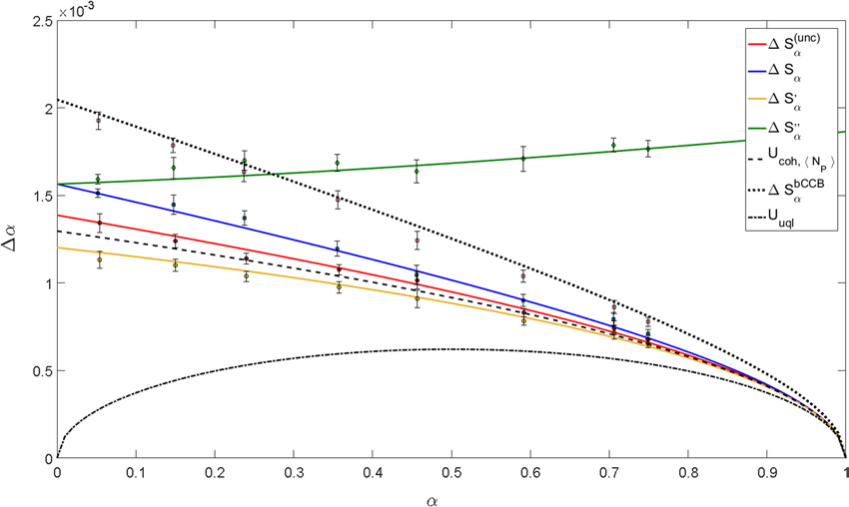
Uncertainty on *α* in function of the mean value of *α*. Four different estimators are considered. Solid lines are the theoretical curves, dashed and dotted lines are the limits corresponding to the best quantum and classical cases when the same mean energy of the probe and the same detection efficiency are considered. The markers are the experimental data. In this configuration the measured efficiencies are *η*_*P*_ = 0.76 and *η*_*R*_ = 0.43 < 0.5, while $\langle {N}_{P}\rangle  \sim 50\cdot {10}^{4}$. In this condition ${\rm{\Delta }}{S}_{\alpha } > {\rm{\Delta }}{S}_{\alpha }^{(unc)}$ while Δ*S*′_*α*_ remains below the classical benchmark.

**Figure 5:**
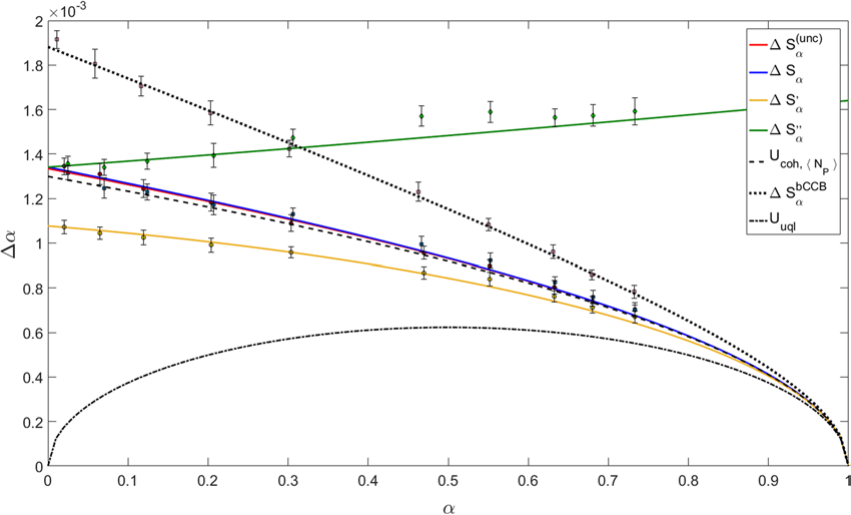
Uncertainty on *α* in function of the mean value of *α*. Four different estimators are considered. Solid lines are the theoretical curves, dashed and dotted lines are the limits corresponding to the best quantum and classical cases when the same mean energy of the probe and the same detection efficiency are considered.The markers are the experimental data. In this configuration the measured efficiencies are *η*_*P*_ = 0.76 and *η*_*R*_ = 0.49, while $\langle {N}_{P}\rangle  \sim 50\cdot {10}^{4}$.

In our experimental situation, since ${S}_{P}={S}_{R}\gg {A}_{coh}$ it follows *η*_*coll*_ → 1 and consequently ${\sigma }_{\gamma }=\frac{1+\gamma }{2}-{\eta }_{R}$. Inverting this relation offers a useful way to measure the total efficiencies (Klyshko heralding efficiency) of the two channels, without the need of comparing with calibrated devices^59^. In the experimental situation corresponding to Fig. 3 we measured *σ*_*γ*_ = 0.24 ± 0.03 and *γ* = 1.006, which implies overall heralding efficiencies *η*_*R*_ = *η*_*P*_ = 0.76, as reported in the caption. The same method has been adopted to evaluate the efficiencies in the other cases, reported in Figs 4 and 5.

In all these figures the mean values of *α* (x-axis) and their corresponding uncertainties Δ*α* (y-axis) have been obtained acquiring 200 frames with the absorbing sample inserted. Repeating each measurement 10 times the error bars have been estimated. In particular, for each frame, we integrate the data on *S*_*R*_ and *S*_*P*_, opportunely corrected for the background, obtaining *N*_*R*_ and *N*′_*P*_, necessary for the estimation of the mean absorption *α* according to the different estimators considered, in Eqs (1–16).

To reproduce the single-mode classical strategy we performed a calibration measurement without the sample obtaining 〈*N*_*R*_〉; we then estimate *α* as:

19 $${S}_{\alpha }^{(unc)}=1-\gamma \frac{{N}_{P}^{^{\prime} }}{\langle {N}_{R}\rangle }.$$

For ideal Poissonian statistics of the probe, this strategy leads to the classical lower bound *U*_*coh*_ (see Theory section). In our experiment, the Poissonian behavior is guaranteed by the condition $\mu \ll 1$, as discussed before.

Finally to reproduce the bCCB case we consider a different region of the detector ${S}_{R}^{^{\prime} }$, displaced from *S*_*R*_ and only classically correlated with *S*_*P*_.

Note that from the calibration measurement also *γ*, *σ*_*γ*_, *F*_*P*_ and *F*_*R*_ can be simply evaluated.

## Results and Discussion

**Figure 6:**
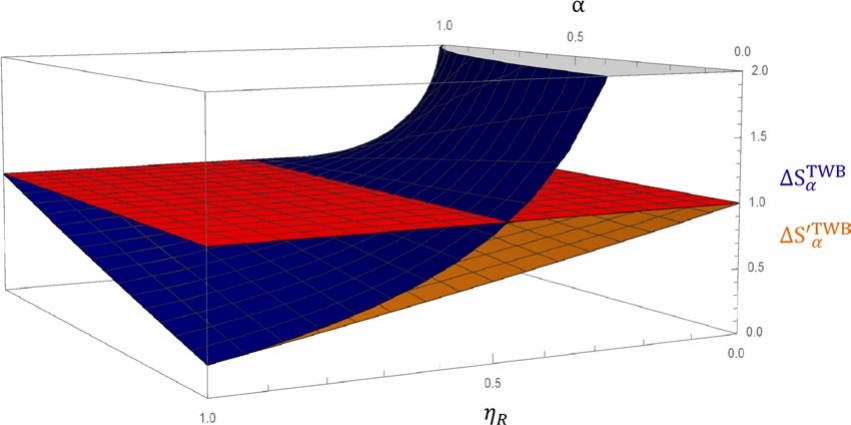
Uncertainty on *α*, normalized to the single mode coherent case (${U}_{coh,\langle {N}_{P}\rangle }$, red surface), using TWB as input state and the two different estimators presented in the text (*S*_*α*_ in Eq. (1), blue surface, and *S*′_*α*_ in Eq. (13), orange surface) in function of the losses on the reference path, *η*_*R*_, and *α*. It turns out that for *η*_*R*_ close to one ${\rm{\Delta }}{S}_{\alpha }^{(TWB)} \sim {\rm{\Delta }}{S^{\prime} }_{\alpha }^{(TWB)}$; on the other hand for, *η*_*R*_ < 0.5, ${\rm{\Delta }}{S}_{\alpha }^{(TWB)} > {U}_{coh,\langle {N}_{P}\rangle }$ while ${S^{\prime} }_{\alpha }^{(TWB)}$ always remains below this limit.

In Eqs (11) and (15) we have explicitly reported the uncertainty achieved by TWB for the estimators ${S}_{\alpha }^{(TWB)}$ and ${S}_{\alpha }^{^{\prime} (TWB)}$ respectively, in case of balanced total efficiencies in the probe and reference beam. The unbalanced case leads to cumbersome analytical expressions, so we report this situation graphically in Fig. 6. The uncertainties on these two estimators are compared at varying *η*_*R*_ and fixed *η*_*P*_ with respect to the classical lower bound ${U}_{coh,\langle {N}_{P}\rangle }$, evaluated for the same number of detected photons. It emerges that for *η*_*R*_ = 1 the two estimators offer exactly the same quantum enhancement, maximum for $\alpha \ll 1$. Nonetheless, for *η*_*R*_ ≠ 1 and sufficiently large, the performances of the two estimators remain comparable. Instead when *η*_*R*_ < 0.5 the uncertainty on ${S}_{\alpha }^{(TWB)}$ becomes greater than the classical one; on the contrary ${\rm{\Delta }}{S}_{\alpha }^{^{\prime} (TWB)}$ maintains always below it. Note that in Fig. 6 we fix *η*_*P*_ = 0.76 (the value of our experiment) and we considered the dependence from *η*_*R*_. The opposite situation, where *η*_*R*_ is kept fix is not reported. In this case Δ*S*_*α*_ and Δ${S}_{\alpha }^{^{\prime} }$ behave similarly for all the variability range of *η*_*P*_, and are always below ${U}_{coh,\langle {N}_{P}\rangle }$.

These different regimes at varying *η*_*R*_ have been experimentally explored with our set-up and the results are shown in Figs 3–5. In these figures, considering different estimators, the dependence of the uncertainty on *α* in function of its mean value is reported. The three situations only differ from the value of *η*_*R*_ considered. The solid lines are the theoretical curves in Eqs (9, 5 and 17) and the equivalent of Eq. (14) in the general case of *γ* ≠ 1 where the experimental values of the quantities *σ*_*γ*_, *F*_*P*_, *F*_*R*_, *γ* have been substituted. The markers represent the experimental data which are in a good agreement with our theoretical model describing experimental imperfections. The black curves stand for significant limits, obtained with ideal states. The dotted-dashed line is the fundamental quantum limit *U*_*uql*_ = [*α*(1 − *α*)/〈*n*_*P*_〉]^1/2^, achievable by TWB and unitary efficiencies. The dashed line is the classical lower bound calculated for the actual number of detected photons, ${U}_{coh,\langle {N}_{P}\rangle }$, while the dotted line is the classical limit in the two-mode balanced case, ${\rm{\Delta }}{S}_{\alpha }^{(bCCB)}$. Figure 3 reports also the classical lower bound assuming no losses occurring after the sample, ${U}_{coh,\langle {n}_{P}\rangle }\mathrm{=[(1}-\alpha )/\langle {n}_{P}\rangle {]}^{\mathrm{1/2}}$, where 〈*n*_*P*_〉 is the number of photons of the probe interacting with the sample. This quantity can be easily estimated as $\langle {n}_{P}\rangle =\langle {N}_{P}\rangle {\eta }_{d}^{-1}$, where *η*_*d*_ represents the detection efficiency after the sample. The obtained value of *η*_*d*_ = 0.80 ± 0.01 takes into account transmission and collection losses trough all the optical elements after the sample (a lens, an interference filter and the quantum efficiency of our CCD camera). The efficiency of the camera with the filter placed in front of it has been experimentally measured using the technique presented in^58^ (*η*_*CCD*_ = 0.84 ± 0.01).

Although experimental not unitary efficiencies lead to a remarkable detachment from the UQL, for $\alpha  \sim \mathrm{2 \% }$, we still obtain a significant quantum enhancement: ${U}_{coh,\langle {N}_{P}\rangle }/{\rm{\Delta }}{S^{\prime} }_{\alpha }=1.51\pm 0.13$ and ${\rm{\Delta }}{S}_{\alpha }^{(bCCB)}/{\rm{\Delta }}{S^{\prime} }_{\alpha }=2.00\pm 0.16$. The comparison respect to the classical lower bound assumed with ideal detection efficiency leads to ${U}_{coh,\langle {n}_{P}\rangle }/{\rm{\Delta }}{S^{\prime} }_{\alpha }=1.32\pm 0.14$.

The comparison with the two-mode classical strategy (${S}_{\alpha }^{(bCCB)}$) is of particular interest since the two-beam approach allows compensating unavoidable drifts and instability of source and detectors, leading to an *unbiased* estimation of *α*, i.e. not affected by temporal drifts of the experimental set-up. Estimators *S*_*α*_ and ${S}_{\alpha }^{^{\prime\prime} }$ do not require the knowledge of the individual absolute power of the source or detector response but a measurement of the average arms unbalance in absence of the object $\gamma =\frac{\langle {N}_{R}\rangle }{\langle {N}_{P}\rangle }$, and the condition for having an unbiased estimator is the stability of this parameter. Experimentally, this is much less demanding than controlling the power stability of the individual probe beam (i.e. 〈*N*_*P*_〉 constant over time) and detector response for the direct/single beam case. Indeed, it is expected that the factors affecting the source and the detectors act in the same way on the probe and on the reference channels.

On the other side ${S}_{\alpha }^{^{\prime} }$, in particular the calculation of *k*_*opt*_ and *δE*, requires the knowledge of the two absolute values of both the efficiencies *η*_*R*_ and *η*_*P*_, which include optical transmission and detectors quantum efficiency. The last one is usually obtained by comparison with calibrated radiometric standards. Alternatively, they can be determined from the same SPDC set-up by using some extensions of the Klyshko’s method^58–60^. This second approach is the one used in the present paper: as described after the Eq. 18, absolute arms efficiencies can be extracted from the measured value of *σ*_*γ*_. In any case, uncertainty smaller than 10^−3^ is quite challenging in the calibration of detector operating at low optical power. Inaccuracy in the determination of these parameters, although does not propagate directly to the loss estimation, could somehow affect the optimality of ${S}_{\alpha }^{^{\prime} }$. Furthermore, ${S}_{\alpha }^{^{\prime} }$ could be affected by drift in the mean value of 〈*N*_*P*_〉, as it happens for the single mode strategy.

## Conclusion

We address the question of loss estimation and analyze different measurement strategies. In particular we show that with a simple photon number measurement of TWB state it is possible to approach the ultimate quantum limit of the sensitivity in case of perfect detection efficiency. The experiment reports the best sensitivity per photon ever achieved in loss estimation without any kind of data post-selection. Indeed, as far as we know the best reported result is a quantum enhancement of 1.21 ± 0.02, recently achieved by Moreau *et al*.^27^. Also other transmission based experiments demonstrating significant quantum enhanced sensitivity are present in literature, as^39^, however their results are not directly comparable with ours since the uncertainty on the absorption coefficient is not reported.

In particular we double the sensitivity of the conventional classical two-beam approach and we overtake of more than 50% the sensitivity of the coherent case. The advantage, considering perfect detection efficiency of the classical beam after the sample, reduces to 32%. At the same time these results accurately confirm the theoretical model accounting for experimental imperfections.

The estimator represented by *S*_*α*_ in Eq. (1)^24^, is compared both theoretically and experimentally, with other estimators in literature (see Eqs (13) and (16)) in presence of experimental imperfections (e.g. not unitary detection efficiency). Despite in case of high detection losses the estimator ${S}_{\alpha }^{^{\prime} }$ in Eq. (13) has the smallest uncertainty, it turns out that where the quantum enhancement is significant, i.e. for sufficiently high efficiencies, *S*_*α*_ and ${S}_{\alpha }^{^{\prime} }$ approximately offer the same quantum enhancement. Moreover, we argue that *S*_*α*_, beside its simple form, has several practical advantages. On the one side, it is robust to experimental unavoidable drifts of the sources and detectors, leading to unbiased estimate. On the other side, it does not require absolute detection efficiency calibration. These features are of the utmost importance in view of real applications.
